# Reflective and feedback performances on Thai medical students’ patient history-taking skills

**DOI:** 10.1186/s12909-019-1585-z

**Published:** 2019-05-14

**Authors:** Weeratian Tawanwongsri, Tharin Phenwan

**Affiliations:** 1 School of Medicine, Walailak University, 222 Taiburi, Tha Sala, Nakhon Si Thammarat, 80161 Thailand; 2 School of Nursing and Health Science, University of Dundee, Dundee, DD1 4HJ UK

**Keywords:** Reflective practice, Feedback, History taking, Thai

## Abstract

**Background:**

Reflective practice (RP) plays a crucial role in encouraging learners to think critically and consciously about their performances. Providing constructive feedback can further enhance RP. But non-Western learners might face different learning barriers compared to learners in the West, where RP originated.

**Methods:**

In this retrospective study, we assessed RP and feedback performances on Thai medical students’ patient history-taking skills. We applied RP and peer feedback, along with feedback from the instructors, during the history-taking sessions of the ten-week introduction course for fourth-year medical students. Twelve history-taking sessions were used for the analysis. Two instructors assessed students’ reflective performance and categorised them into one of the six stages of Gibbs’ reflective cycle; their feedback performances were analysed using Pendleton’s model. We investigated the correlations between students’ overall grade point average (GPAX) and patient history-taking scores on the Objective Structured Clinical Examination (OSCE). Students’ opinions of the RP teaching method were also collected.

**Results:**

All (*n* = 48) students participated in our study. The students’ mean age was 21.2 ± 0.5 years. The majority of the students were female (64.6%). The data indicated that 33 and 4% of the participants were categorised into the evaluation stage and action plan stage of Gibbs’ reflective cycle, respectively. In addition, 22 and 15% of the participants were able to state what their peers did well and suggest how peers could improve their skills, respectively. All students passed the minimum passing level of four history-taking OSCE stations. Participants agreed that RP was a useful tool (mean 9.0, SD 0.1), which enhanced their thought processes (mean 8.4, SD 0.2) and future performances (mean 8.2, SD 0.2). However, there was no correlation between the students’ highest Gibbs’ reflection levels and their history-taking OSCE scores.

**Conclusions:**

RP, together with feedback, proved to be a useful technique to help fourth-year Thai medical students improve their reflection skills, enhance their medical knowledge, and improve patient history-taking skills. Further study with longer monitoring is required to further explore negative and positive influential factors affecting students’ achievement of better reflection performances.

**Electronic supplementary material:**

The online version of this article (10.1186/s12909-019-1585-z) contains supplementary material, which is available to authorized users.

## Background

Reflective practice (RP) is a process in which learners consciously reflect and think about their thought processes and actions, critically evaluating them, in order to improve their future performances [1, 2]. One of the most popular theories related to RP is Gibbs’ reflective cycle, which represents a simplified process within the RP context and consists of six stages: description, feelings, evaluation, analysis, conclusion, and action plan [3]. This method helps learners achieve a higher level in their thought processes and also enhances their lifelong learning skills [4]. Some students might develop their capabilities independently [5]. However, this process can be greatly enhanced through well-trained educators’ facilitation of their performances by using constructive feedback [6, 7]. For example, facilitators can give students structured guidelines, such as “what”, “why”, and “what they could have done differently” [6]. If trained properly and efficiently, learners would be more adaptable in various situations that require reflexivity [8]. RP learners develop better metacognition and lifelong learning skills associated with better long-term patient care [9–11]. This method can also improve leadership capacity and increase learners’ competency in non-biological domains, such as collaboration skills [12, 13]. Thus, doctors who lack the ability to maintain their reflection skills and adjust them in real-life situations often have poorer insights into their performance than those with such skills [14].

Previous studies have demonstrated that RP could enhance several aspects of desirable outcomes in the medical curriculum, such as professionalism, patient safety, palliative care, clinical communication, and clinical skills [15–21]. This applies to other fields outside medicine as well. For example, Williams and Wessel found that 25% of physical therapy students’ reflective thinking at McMaster University reached the top level of reflection (i.e., ‘indicates future behaviour’) [22]. They could clarify the issues, develop their skills, and solve similar problems in the future.

Despite the effectiveness of RP, most studies have evaluated the students’ RP performance using written assignments [8, 19, 21, 23]. The process was found to be time- and human resource-consuming and was less enjoyable for the instructors than working directly with students [24, 25]. In addition, no study has yet assessed medical students’ thought processes compared to using Gibbs’ reflective cycle. Evidence of RP’s effectiveness in non-Western countries, where learners might face barriers or differences such as different teaching styles, is also lacking. For example, Thai educators have emphasised the use of feedback for correction while educators in other cultures have been reluctant to give the feedback due to the concern of negative emotional consequences [26]. Another difference relates to social structures; educators and doctors in Thailand are at the very top tier of society, so students are unlikely to challenge their teachers [27]. Moreover, although medical schools in Thailand use the same curriculum, there are lots of variations in the teaching methods.

From what we have observed, our medical students found it difficult to express their opinions or different arguments from their peers in public due to the fear of losing face, or the possibility of breaking the junior–senior (big person/little person) relationship with their teachers, thereby causing them unnecessary discomfort [27].

In addition to RP, feedback is also an essential tool that drives improved clinical performance [28, 29]. Pendleton’s rule is one of a common methods for providing feedback in medical education [30]. It helps to promote and fulfil self-reflection particularly on the development of learners’ practical skills [31, 32]. According to this model, trainers need to state what the learner did well and recognise areas for improvement as well as how to achieve an acceptable level of competency [31]. In learners’ future career settings, peer feedback will be the only tool they have for achieving this in order to provide effective patient care. While the reliability of learners’ peer assessments has not been well established [33], the effective feedback from experienced and trained instructors is considered as a crucial tool to enhance the capacity of the learners. The feedback from trained instructors will help learners to self-reflect on their practice in order to reach the acceptable level of competency [31, 34].

In our medical school, when students enter their fourth year, they must complete a ten-week course entitled Introduction to Clinical Medicine, during which the concepts of RP and feedback are introduced. However, there has been no objective assessment of the course’s effectiveness on RP teaching at all. This study asks the following research question: By using Gibbs’ reflective cycle as a framework, how effective are RP and the peer feedback teaching method used in this course for helping our students develop these skills and enhance their learning? The primary objective is to assess the reflective and feedback performances on Thai medical students’ patient history-taking skills. The secondary objective aims to assess the correlation between students’ highest Gibbs’ reflection levels and their history-taking Objective Structured Clinical Examination (OSCE) scores as well as investigate students’ opinions of the RP teaching method.

## Methods

### Context

Our school of medicine offers a six-year MD programme for Thai students, admitting 48 students each academic year. In the first three years, called the pre-clinical years, students study basic sciences; in the last three years, the clinical years, they learn clinical science and start their hospital rotations. The most critical transition period is usually at the beginning of their fourth year, when students move from the pre-clinical learning environment to the clinical setting. For this study, students were divided into five groups of nine or ten students each. Students were mixed and balanced among groups according to their academic achievements.

### Teaching method

Once students reach their fourth year, they enrol in a mandatory ten-week course entitled Introduction to Clinical Medicine, in which the concept of RP is introduced. The course includes history-taking sessions and simulated patients (SP), physical examination sessions, procedural skill sessions, and exposure to real patients at affiliated hospitals. In this course, one of the learning objectives is that students learn to take a comprehensive patient history.

The course started with both instructors (TP and WT) explaining and demonstrating the step-by-step concept of RP to the students. The students then engaged in hands-on practice with two mock history-taking sessions. RP was used in the history-taking sessions because these sessions are cognitive based whereas the other parts of this course are a mixture of procedural competency and professionalism. We assigned 15 different chief complaints for the history-taking practices. In each session, one student was randomly selected to take the history-taking with a SP with one specific chief complaint in a separate room. Meanwhile, other students and the instructors would observe the session via a closed-circuit television (CCTV) in the lecture hall. Each history-taking session lasted for twenty to thirty minutes. Afterwards, the interviewer would come to the front of the classroom to reflect on his or her performance (i.e., engage in individual RP). After that, each group would conduct a five-minute group discussion while the interviewer waited for peers’ feedback. Five volunteers (one from each group) then gave feedback to the interviewer, which lasted approximately thirty minutes. Following Pendleton’s rule, we encouraged the peers to state what the interviewer did well and recognise areas for improvement as well as how to achieve them. The social constructionism paradigm was also used as the framework to improve students’ reflective process. This paradigm *“tend [s] to move learners out of their ‘comfort zone’ to make collaborative ‘sense’ of potential skills being presented to them”* [35].

If the students struggled to perform their RP or give any feedback, the instructors would guide them using probing questions: 1) How was your peer’s history-taking performance? 2) What did they do well? In what ways was it well? 3) What else could be improved? How would you do it otherwise? The second interviewer then entered the interview room to repeat the entire process. There were two rounds with two different SP on each day (i.e., ten students were selected for the RP in each session). All sessions were video-recorded and distributed as learning materials for the students to review their performances. The summary of the whole process can be found in the Additional file 1: Fig. S1).

### Course and instructors’ validation

The course was initially designed by an expert at our medical school and later adapted by the authors. Both authors attended a comprehensive course about how to guide students with reflection and feedback; the course was taught by a medical education expert in the school of medicine. All guided questions and the teaching process were discussed thoroughly and approved by the expert before we began this course.

### Data collection and evaluation

This retrospective study used random sampling without a control method. We collected the participants’ baseline characteristics, including age, gender, and GPAX. At the end of each session, the two instructors individually categorised the students’ best performance into each level of Gibbs’ cycle using a constant comparative method (i.e., the authors assigned a Gibbs’ level to each student). We (TP and WT) selected Gibbs’ model of reflection because of its practical questions to determine each participant’s reflection cycle level [36]. For example, if a student only described the session without any clear reflection, they would be categorised in the “description” level. We also evaluated peers’ feedback as mentioned in Pendleton’s rule. Any discrepancies were thoroughly discussed between instructors before making a final decision on which levels of reflection and clinical performance scores were appropriate based on participants’ performances. The instructors also watched the sessions again to ensure that the categorisation was final. Three out of fifteen sessions were not available due to technical errors, resulting in a total of twelve sessions being used for the final analysis. In total, there were twenty-four individual RP events (two students from each RP session for twelve sessions). To assess their academic outcome, participants’ examination score from four patient history-taking scores in the OSCE was collected and compared with their Gibb’s cycle. A minimum passing score of 60% with criterion-referenced grading set by the curriculum committee of the school was required to pass each OSCE station. Students’ anonymised feedback regarding this teaching method was also collected after the course. The survey used self-reported scores with a visual analogue scale (VAS) of 0–10, with a space for extra comments.

### Statistical analysis

For the qualitative analysis, the instructors assessed participants’ highest reflective stage and categorised them according to Gibbs’ learning cycle using a constant comparative method by looking at and analysing their thought processes during the session and the VDOs. We also counted the students able to state what the learner did well and recognise areas for improvement as well as suggest ways to do so. Mean and standard deviation (SD) or median and range were used to describe the continuous data. Frequency and percentages were used for categorical data. We used a regression analysis to find the correlation between end-session reflection performances and history-taking OSCE scores. A *p* value of < 0.05 in two-tailed tests was considered statistically significant. The statistical analysis was performed using SPSS software version 17 (SPSS Inc., Chicago, IL, USA).

## Results

All fourth-year medical students participated in our study (*n* = 48). The participants’ mean age was 21.2 ± 0.5 years. The majority of participants were female (*n* = 31, 64.6%). We collected and evaluated the reflective performances from 24 students (24 reflection sessions) and the feedback performances from 48 students (117 feedback sessions). The mean GPAX of male and female students were 3.42 ± 0.30 and 3.43 ± 0.27, respectively. Based on the Gibbs’ reflection model, students’ performances were categorised into six stages. Their reflective stages together with verbatim quotes are shown in Table 1.

**Table 1:** Gibbs’ reflection model and examples of students’ reflection quotes

Stage	Elaboration	Verbatim quotes
Description	Participant described what transpired within the room without any meaningful interpretation.	***“*** *He [the interviewer] dressed well and used simple language with the patient.” Student 2/session 1*
Feeling	Participant described what he/she felt or his/her thought processes.	***“*** *Nerve-wracking.* ***”*** *Interviewer 1/session 1*
Evaluation	Participant reflected on the good parts or parts that could be improved without any tangible details.	*“I did well on the past history part. But the present illness was not clear.” Interviewer 2/session 2*
Analysis	Participant reflected on the good parts or parts that could be improved with details.	*“The review of system part was non-sequential, not head-to-toe.” Student 3/session 3*
Conclusion	Participant reflected on the good parts or parts that could be improved and suggested areas for the next session without concrete examples.	*“I will probe more about the fever pattern next time.” Interviewer 2/session 4*
Action plan	Full description of the good parts and parts that could be improved with concrete suggestions for the next session.	***“*** *Next time, I will ask more details about the volume status caused by diarrhoea by asking about how much volume has been lost. I may compare the volume with a glass of water to make it more objective.” Interviewer 1/ session 10*

**Fig. 1:**
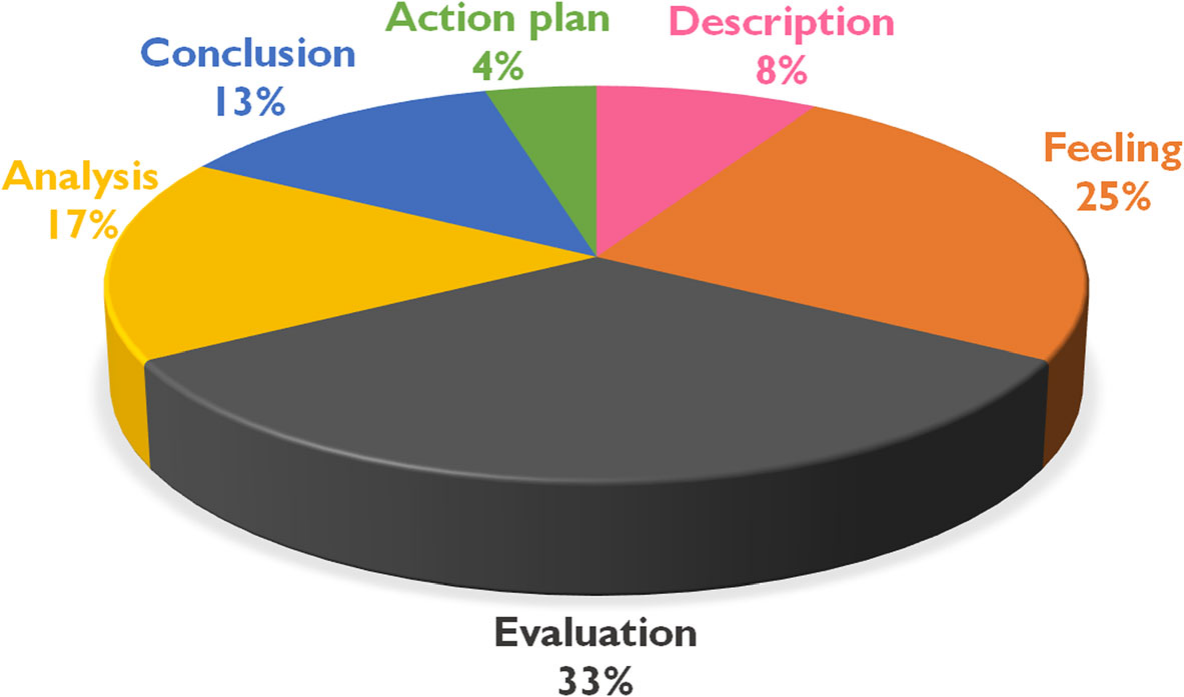
Reflection performance related to the Gibbs’ cycle (*n* = 24). Participants’ performances were evaluated and categorised into each level of Gibbs’ cycle using a constant comparative method. These six stages include description, feelings, evaluation, analysis, conclusion, and action plan

For the individual RP sessions, 33% of participants reached the evaluation level shown in Fig. 1. Only 4% of students reached the highest desirable outcome (i.e., the action plan stage). For the feedback sessions, 22 and 15% of participants were able to state what the peers did well and suggest how to improve their skills, respectively.

**Fig. 2:**
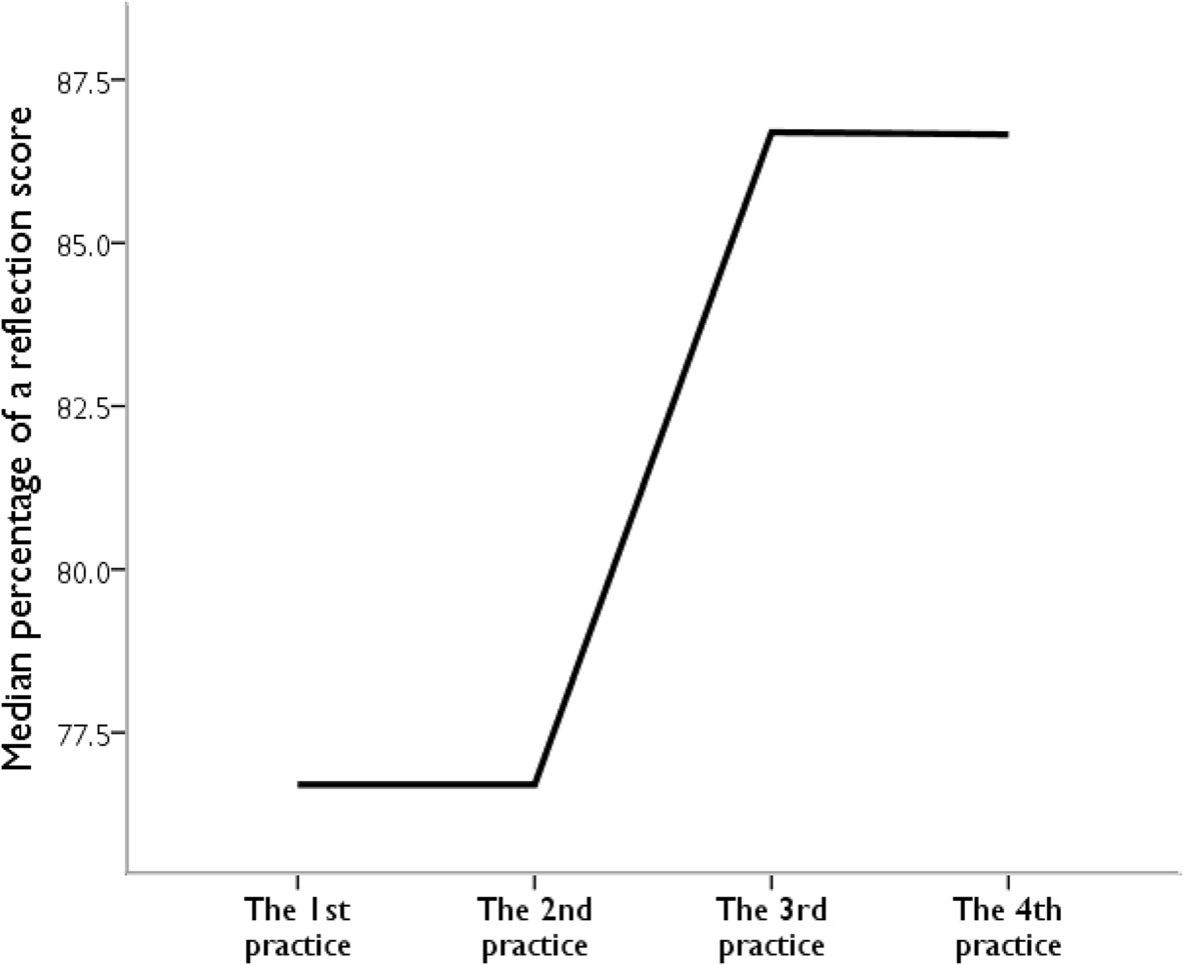
Changes in clinical performance scores during weekly evaluation (*n* = 48). The participants’ performances were assessed by the instructors using a visual numeric scale from 1 to 10 for each domain. These domains included medical knowledge, professionalism, as well as, interpersonal and communication skills

We found a correlation between participants’ GPAX and their clinical performance scores, r (48) = + 0.36, *p* = 0.05; clinical performance scores and history-taking OSCE scores, r (48) = + 0.36, *p* < 0.05; and GPAX and history-taking OSCE scores, r (48) = + 0.56, *p* < 0.05. The median percentage of clinical performance scores significantly increased in the third practice (*p* = 0.03); after that, it remained unchanged, as shown in Fig. 2.

At the end of the course, all participants passed the minimum level of the four history-taking OSCE stations. However, no correlation was found between students’ highest Gibbs’ reflection levels and their history-taking OSCE scores.

We collected students’ anonymised feedback regarding this teaching method. The survey was self-reported scores with a VAS of 0–10 and an extra comment section, as shown in Table 2. The students agreed with a usefulness of reflective practice with the mean score of 9.0 ± 0.1. They rated their level of agreement on the topics ‘reflective practice helps me prepare my studies in advance effectively’ and ‘reflective practice enhances my critical thinking skills based on the clinical reasoning’ with a mean score of 8.2 ± 0.2 and 8.4 ± 0.2, respectively. They also rated their level of agreement on the topic ‘I enjoyed this course’ with a mean score of 8.5 ± 0.2.

**Table 2:** Students’ opinions of the reflective practice teaching method (*n* = 48). They rated their level of agreement on a 10-point scale for each topic as shown below

Topics	Mean	SD
Reflective practice is a useful tool	9.0	0.1
Reflective practice helps me prepare my studies in advance effectively	8.2	0.2
Reflective practice enhances my critical thinking skills based on the clinical reasoning	8.4	0.2
My reflection skill before the course	5.1	0.3
My reflection skill at the end of the course	8.0	0.3
I enjoyed this course	8.5	0.2

For the written part of the survey, thirteen participants gave positive feedback regarding this teaching method. Two participants wrote that they felt pressured and stressed at the beginning of the course, mainly because they were unprepared and feared being publicly humiliated or becoming an object of scrutiny by their peers or instructors. They also stated that it was better after a few sessions.

## Discussion

RP is one learning method that enhances students’ critical thinking and reflection skills—key skills for the twenty-first-century learning skills framework. Studies have shown the effectiveness of RP, especially among adult learners and medical students, who need flexibility in their performance [8, 13]. Our work is the first study to focus on evaluating the effectiveness of RP teaching methods in Thai medical students.

We found that only a minority of participants could reach the highest stage of Gibbs’ reflective cycle (i.e., action plan) with their individual RP. We will discuss our results from a cultural perspective as the Thai culture strongly differs from western cultures where the RP and feedback were developed. In general, Thais prefer to collaborate as a team rather than work as individuals. We are more collective and often display high uncertainty avoidance, meaning that we do not accept changes easily [37]. We are not confrontational by nature and find it difficult to express our thoughts publicly due to the fear of losing face [27]. When changes occur, they usually happen within a group rather than being carried out by an individual [37, 38].

This cultural background is consistent with our findings that a small percentage of the students (4%) reached the action plan level compared to a previous study of physiotherapist students (25%) [22]. Besides the cultural background, there are several plausible explanations for this finding. First, there were differences in the teaching methods, teachers’ skills, and participants’ characteristics between these two contexts. The course design was also different. Second, as RP was introduced as a new concept during the beginning of students’ fourth year, and it was very likely that participants still lacked experience in RP at this time. This explanation concurs with another study that postgraduate students were more likely to have deeper forms of reflection than undergraduate students [39]. Thus, continuous stimulation to use RP to enhance students’ skills is recommended. Third, some participants admitted that they felt shy and afraid of expressing their thoughts publicly. This issue is also linked to the Thai culture, which hinders the expression of public opinion, especially to those in a more senior position. The course was designed so that students had to express themselves in front of the whole class and instructors. This design may be the source of their discomfort. Written reflective writing along with oral RP may help mitigate this challenge [21–25]. Previous studies have also reported that collaborating in teams, for instance, as in the team-based learning (TBL) approached resulted in better performance than individual learning [40]. TBL is also another beneficial learning method, as recommended in the meta-analysis [41], as this approach is based on constructivist learning theory that students can learn with facilitators to guide their different understandings [42]. Our study also revealed that peers were able to recognise areas for improvement and suggest action plans at a higher rate (15%) than individual RP (4%). The low percentage of achievement may imply that students, particularly beginning learners, need feedback from either peers or experienced instructors in order to improve their future performance because feedback helps promote and fulfil self-reflection [31, 32].

Another noteworthy finding is the lack of correlation between students’ highest Gibbs’ reflection levels and their history-taking OSCE scores. This may stem from the foundation objective of OSCE, as it aims to assess multiple dimensions of clinical skills developed through repetitive practices or long-term experiences. To assess students’ RP objectively, a longitudinal assessment—like the one in this study—may be preferable using the well-designed OSCE checklist with acceptable validity and good inter-observer reliability [43].

Our work is the first study to evaluate medical students’ RP and link it to Gibbs’ reflective cycle. The categorisation of the findings also made it more practical to determine their learning outcome clearly. The two instructors individually analysed and categorised the findings to ensure that the data were more rigorous. Our study demonstrated that RP can be taught to non-Western students after customising it to the learners’ culture so that they would feel comfortable enough to express their thoughts. Although not many of the participants in this study reached the highest stage of the Gibbs’ cycle (i.e., the action plan), it might be due to the fact that they were still in the early stages of their RP learning journey.

Our work has several limitations. Firstly, students’ RP was assessed during a short period (i.e., ten weeks). A longer observation or a regular assessment over time might yield additional data regarding their RP. Moreover, their professionalism could not be effectively assessed using a competency-based approach, especially in this short timeframe. Secondly, there may be a potential link between students’ RP and peer feedback skills. Thus, further data collection is required to determine the correlation of the RP and feedback skills. Thirdly, this study did not compare this method in a western culture. Therefore, further comparative studies concerning culturalaspeccts are still needed.

Furthermore, although two instructors served as the main evaluators, several untrained staff also joined the course intermittently. During many sessions, ineffective and/or inappropriate feedback that unfortunately created an unsafe learning environment was generate. For example, some comments were an attempt to shame the students publicly and provide patronising feedback by the use of foul language to dehumanise them in front of their peers. Thus, future courses should include a clear understanding of the course objective and make RP explicitly clear to any extra staff members because these courses help medical students focus on their reflective process, not achieve the end result (e.g., a correct diagnosis). The other alternatives are to adjust the course to use only trained instructors to avoid such unfavourable events altogether. Finally, we also did not have a control group, so we could not determine how the students would progress in each session without RP.

### Implications of the study

Based on our findings, we propose that instructors with good facilitating skills who can build positive and supportive relationships with students are crucial. In a safe environment and with proper guidance, students can develop their skills to achieve their full potential. Cultural differences are another critical factor that educators should consider. For example, in our context, learning and practising learners’ reflection as a group may be more beneficial to the students than as an individual activity. We may not be able to generalise our findings or give a comprehensive explanation of this aspect, but our data and previous findings indicate that the safe learning environments together with useful guidance and the well-designed course—which is compatible with our cultural context—are the critical factors affecting our students’ learning experience. Thus, any interventions or teaching methods that aim to improve students’ learning experiences need to be tested and contextualised as well. Finally, RP should be applied throughout the course as well as be stimulated and assessed regularly so that students can achieve a higher level of reflection in the future.

## Conclusion

RP, together with feedback, proved to be a useful technique to help fourth-year Thai medical students improve their reflection skills, enhance their medical knowledge, and improve their patient history-taking skills. As beginner learners, students may need feedback to help them achieve more favourable reflective performance and the highest desirable outcome (i.e., the action plan level). Further study with longer monitoring is required to further explore the negative and positive factors influencing students in reflecting more effectively.

## Additional file

Additional file 1: **Figure S1.** Learning organization and data collection. Each step was illustrated with pictures together with a concise description as follows. (DOCX 243 kb)

## Abbreviations

CCTV: Closed-circuit television
GPAX: Overall grade point average
OSCE: Objective Structured Clinical Examination
RP: Reflective practice
SP: Simulated patients
TBL: Team-based learning
