# Supplementary material for: Reflective and feedback performances on Thai medical students’ patient history-taking skills
Source: BMC Med Educ. 2019 May 14;19:141. doi: 10.1186/s12909-019-1585-z (PMC6518691; doi:10.1186/s12909-019-1585-z)
Supplement: Supplementary file 1 — Figure S1. Learning organization and data collection. Each step was illustrated with pictures together with a concise description as follows. (DOCX 243 kb) [file 12909_2019_1585_MOESM1_ESM.docx]

Additional file 1

Figure S1. Learning organization and data collection. Each step was illustrated with pictures together with a concise description as follows.

| 1. Students were divided into five groups comprised of 9-10 students within each group. | 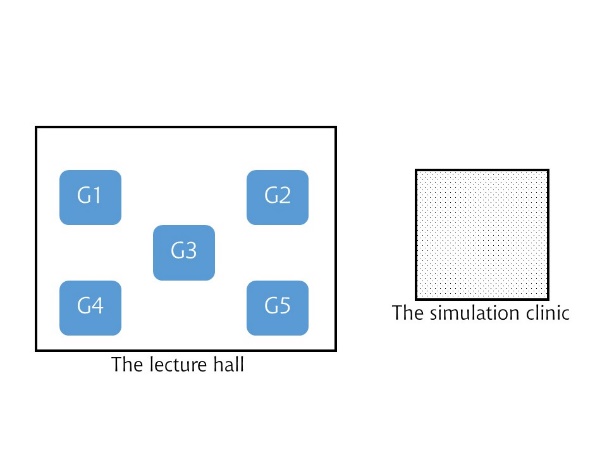 |
| --- | --- |
| 1. One student was randomly selected as an interviewer for taking a history from a simulated patient with given chief compliant in the simulation clinic. | 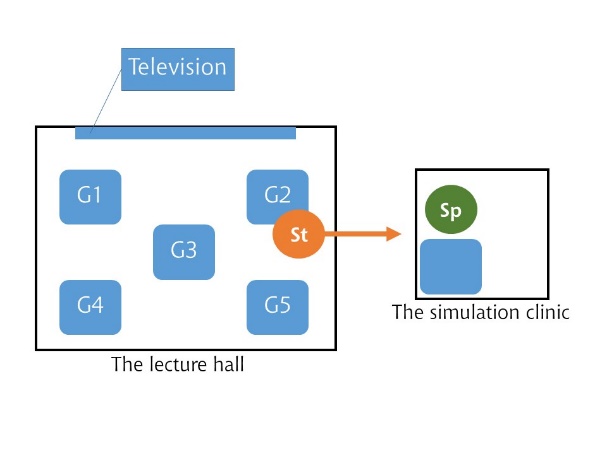 |
| 1. While the interviewer was taking a history, the other students and instructors observed the session via a closed-circuit television in a lecture hall. | 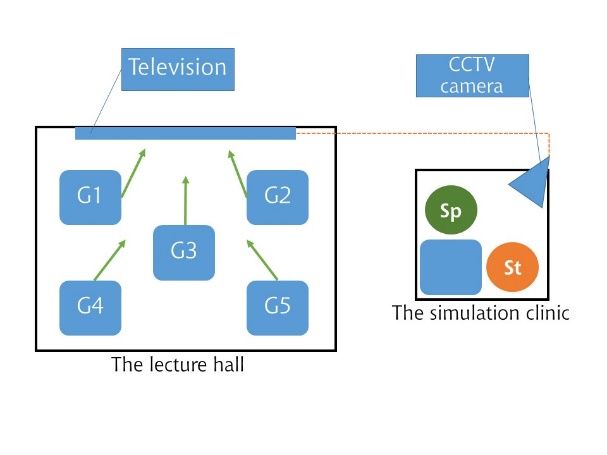 |
| 20-30 minutes | |
| 1. The interviewer came back to reflect his or her own performance in front of the lecture hall—individual RP. | 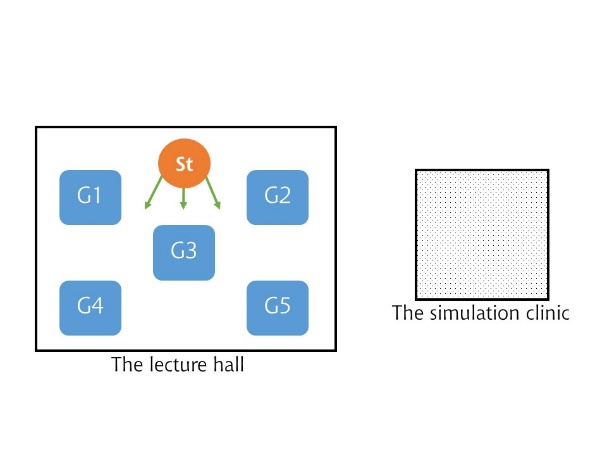 |
| 5 minutes | |
| 1. After 5-minute group discussion without the interviewer while the interviewer was still in the room, the other five volunteers (one from each group) embarked on collaborative RP. | 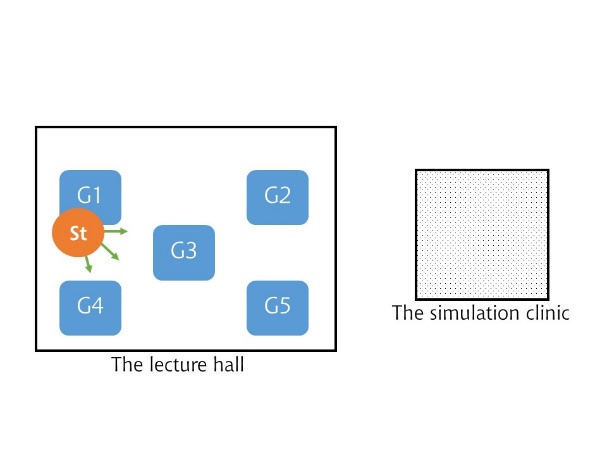 |
| 30 minutes | |
| 1. The instructors provided the feedback (5-10 minutes). | 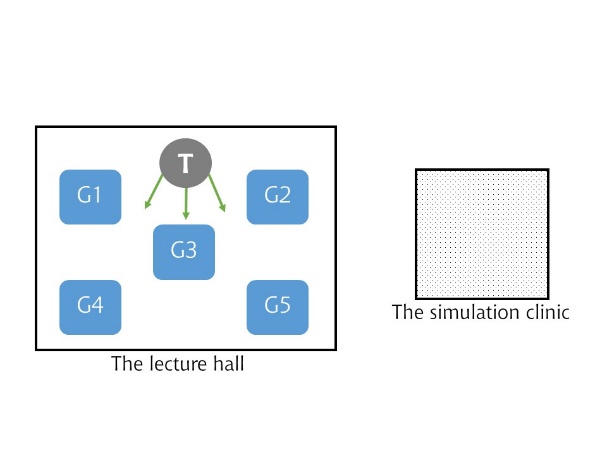 |

Note.—G = Group, Sp = Simulated patient, St = Student, T = Instructor, CCTV = Closed-circuit television. Both individual RP and collaborative RP were all video-recorded and distributed as learning materials for the students to review their own performances. All video files were, thereafter, gathered and analysed to assess the learning outcomes.
